# Indicators of suboptimal response to anti-tumor necrosis factor therapy in patients from China with inflammatory bowel disease: results from the EXPLORE study

**DOI:** 10.1186/s12876-021-02074-z

**Published:** 2022-02-04

**Authors:** Ji Li, Zhanju Liu, Pinjin Hu, Zhonghui Wen, Qian Cao, Xiaoping Zou, Yan Chen, Yingde Wang, Jie Zhong, Xizhong Shen, Dirk Demuth, Olga Fadeeva, Li Xie, Jun Chen, Jiaming Qian

**Affiliations:** 1 Department of Gastroenterology, Peking Union Medical College Hospital, Chinese Academy of Medical Sciences, Shuaifuyuan 1, Dongcheng, Beijing, 100730 China; 2 Shanghai Tenth People’s Hospital of Tongji University, Shanghai, China; 3 The Sixth Affiliated Hospital, Sun Yat-sen University, Guangzhou, China; 4 West China Hospital, Sichuan University, Chengdu, China; 5 Sir Run Run Shaw Hospital, Zhejiang University School of Medicine, Hangzhou, China; 6 Nanjing Drum Tower Hospital, Nanjing University School of Medicine, Nanjing, China; 7 The Second Affiliated Hospital of Zhejiang University School of Medicine, Hangzhou, China; 8 The First Affiliated Hospital of Dalian Medical University, Dalian, China; 9 Ruijin Hospital, Shanghai Jiao Tong University School of Medicine, Shanghai, China; 10 Zhongshan Hospital of Fudan University, Shanghai, China; 11 Takeda Pharmaceutical International AG, Singapore, Singapore; 12 Takeda (China) International Trading Company, Beijing, China

**Keywords:** Anti-tumor necrosis factor, China, Inflammatory bowel disease, Sub-optimal response

## Abstract

**Background:**

Prevalence of inflammatory bowel disease (IBD) is increasing in China. The EXPLORE study evaluated the incidence and indicators of suboptimal responses to first-line anti-tumor necrosis factor (TNF) in patients with ulcerative colitis (UC) or Crohn’s disease (CD). We present results for the mainland China subgroup.

**Methods:**

A retrospective chart review was performed in adults with IBD at 10 centers in mainland China who initiated anti-TNF therapy between 01 March 2010 and 01 March 2015. The cumulative incidence of suboptimal response to first-line anti-TNF therapy was assessed over 24 months using the Kaplan–Meier method. Indicators of suboptimal response were: dose escalation, discontinuation, augmentation with non-biologic therapy, or IBD-related surgery/hospitalization. At site initiation, a survey was conducted with participating physicians to identify barriers to anti-TNF use.

**Results:**

Of 287 patients (72% male) examined, 16/35 (45.7%) with UC and 123/252 (48.8%) with CD experienced a suboptimal response to first-line anti-TNF therapy at any point during the observation period (median 27.6 and 40.0 months, respectively). At 1 and 2 years post anti-TNF initiation, the cumulative incidence of suboptimal response was 51.4% and 75.7% for UC and 45.4% and 57.0% for CD, respectively. Median time to first suboptimal response was 7.2 months for UC and 14.3 months for CD. The most frequent indicator of suboptimal response was discontinuation of anti-TNF therapy (9/16, 56.3%) for UC and IBD-related hospitalization for CD (69/123, 56.1%) followed by augmentation with non-biologic therapy for both cohorts (5/16, 31.3% for UC and 28/123, 22.8% for CD). Dose escalation was the least frequent indicator of suboptimal response to anti-TNF therapy (CD: 4/123, 3.3%; UC: not cited as an indicator). The cumulative incidence of suboptimal response within 4 months of first-line anti-TNF therapy (primary non-response) was over 30% in both cohorts. Financial reasons and reimbursement were identified by surveyed physicians as the most common barriers to prescribing an anti-TNF therapy.

**Conclusions:**

Over one-half of patients with IBD are at risk of experiencing a suboptimal response to first-line anti-TNF therapy at 2 years post-initiation in China. This study highlights a substantial unmet need associated with anti-TNF therapies in China. (Clinicaltrials.gov identifier: NCT03090139).

**Supplementary Information:**

The online version contains supplementary material available at 10.1186/s12876-021-02074-z.

## Introduction

Inflammatory bowel disease (IBD), which includes ulcerative colitis (UC) and Crohn’s disease (CD), is a chronic, relapsing disease of the gastrointestinal tract [1]. While the burden of IBD is greatest in North America and Europe, the incidence of IBD is rapidly increasing in newly industrialized countries in Asia [2]. This includes China, where the crude incidence of IBD is increasing at a faster rate than other countries in Asia, ranging from 0.54 to 3.44 per 100,000 individuals in 2011 [3, 4]. While the age-standardized incidence of IBD in China remains about one-third of that in the US, the growing burden of disease, particularly if it is not well managed, presents a significant challenge for the Chinese healthcare system [2]. This observation is most likely related to urbanization and environmental and lifestyle changes; however, an increased awareness of IBD may also be contributing to this changing pattern [5].

Anti-tumor necrosis factor (anti-TNF) therapy was the first class of biologic therapy approved for patients with moderate-to-severe IBD or who have had an inadequate response to conventional therapy, such as aminosalicylates, corticosteroids, or immunomodulators [6–8]. However, more than half of patients with IBD either do not respond to anti-TNF therapies (primary non-response), or lose response over time (secondary loss of response) within 2 years of initiating treatment [7, 9]. Therefore, these patients often require dose escalation or discontinuation, switching, non-biologic therapy initiation or escalation, or surgery [10]. These indicators of suboptimal therapy were found to be common in patients with IBD in Western nations [7]. In China, infliximab was the only approved anti-TNF therapy for patients with CD until December 2019, and data on the outcomes of infliximab use in IBD from China are limited.

The EXPLORE study assessed the incidence and indicators of suboptimal response to first-line anti-TNF therapy in patients with IBD in real-world clinical practice across 10 newly industrialized countries and regions across Asia–Pacific, Latin America, Russia and the Middle East [5]. This subgroup analysis further investigates the characteristics of patients with IBD in mainland China and describes their response to anti-TNF therapy. Local barriers to prescribing anti-TNF therapy were also investigated.

## Methods

### Study aim, design and data collection

This study aims to assess the incidence and indicators of suboptimal response to first-line anti-TNF therapy in patients with IBD, and the treatment practices of physicians, in 10 hospitals in China as part of the EXPLORE study. Full methodological details have been published previously by Yamamoto-Furusho et al. [5]. Briefly, the EXPLORE study (Clinicaltrials.gov identifier: NCT03090139) was a multinational, retrospective chart review study of adults (≥ 18 years) with IBD who initiated anti-TNF therapy between 01 March 2010 and 01 March 2015 (defined as the index date), and were observed for a minimum of 2 years, and up to 5 years. Patients with an intermediate/unspecified type IBD, who were part of an IBD-related clinical trial during the observational period, received an anti-TNF therapy for any non-UC or non-CD condition or anti-TNF therapy that was outside of the labelled dosing regimen (e.g., episodic administration), had undergone a total colectomy, or whose medical records were unavailable were excluded from the study.

Data from patients residing in mainland China were extracted from either paper or electronic medical records by site personnel across 10 study sites, and information on demographics, medical history, disease location, behavior, and medication history were recorded. Disease activity was primarily based on the closest assessment within 6 months prior to the index date of any endoscopic measurement, if available, or of any documented measurement of full Mayo Score [11] (UC: 0–2 normal, 3–5 mild, 6–10 moderate, 11–12 severe), partial Mayo Score [12] (UC: 0–1 normal, 2–4 mild, 5–7 moderate, > 7 severe), Crohn’s Disease Activity Index [13] (CD: < 150 normal, 150–219 mild, 220–450 moderate, > 450 severe), Harvey-Bradshaw Index [14] (CD: 0–4 normal, 5–7 mild, 8–16 moderate, ≥ 16 severe) or Physician’s Global Assessment [11] (0 normal, 1 mild, 2 moderate, 3 severe). Biochemical activity was based on the closest assessment within 6 months prior to the index date of C-reactive protein (active if ≥ 5 mg/L), albumin (active if < 3.5 g/dL) or fecal calprotectin (active if ≥ 250 mg/kg).

As part of the EXPLORE study, a physician survey was also conducted between June 2017 and June 2018 to review physicians’ experience in managing patients with IBD, including barriers to prescribing anti-TNF therapies and perceived patient challenges in receiving anti-TNF therapies. Participating physicians at selected study sites (all 10 sites in China were selected) were asked to complete a single electronic questionnaire at the time of study initiation. A copy of this questionnaire is included in Additional file 1: Appendix 1.

The study was conducted in accordance with local regulatory and local ethics committee approvals in China. Written informed consent was obtained for all living patients at The First Affiliated Hospital of Dalian Medical University and Zhongshan Hospital Fudan University. A waiver of informed consent was granted by the local ethics committee at the remaining eight sites (Peking Union Medical College Hospital; The Sixth Affiliated Hospital of Sun Yat-sen University; Sir Run Run Shaw Hospital, Zhejiang University, School of Medicine; West China Hospital, Sichuan University; No. 10 People's Hospital of Shanghai; Ruijin Hospital Shanghai Jiao Tong University School of Medicine; The Second Affiliated Hospital of Zhejiang University School of Medicine; and The Affiliated Drum Tower Hospital of Nanjing University) because data were obtained by a retrospective chart review. The study protocol also conformed to the ethical guidelines of the 1975 Declaration of Helsinki as reflected by *a priori* approval by each institution’s human research committee.

### Study outcomes and statistical considerations

The primary outcome was the incidence of suboptimal response to first-line anti-TNF therapy, with the current subgroup analysis focusing on patients from mainland China with UC or CD. Suboptimal response was defined as experiencing ≥ 1 of the following events: anti-TNF dose escalation (any increase in dose and/or frequency of anti-TNF therapy due to non-response that occurs > 4 months after the start of treatment); augmentation with non-biologic therapy (starting or increasing the dose and/or frequency of a concomitant non-biologic therapy due to non-response); discontinuation of anti-TNF therapy (including switching to another anti-TNF therapy within 2 months of discontinuation due to non-response, but excluding events resulting from clinical improvement, adverse events, patient choice or reimbursement issues); or IBD-related surgery (colectomy, ileocolectomy, ostomy, fistula repair, abscess repair, or strictureplasty) or hospitalization (admission for reasons related to non-response/worsening of disease and with a stay of ≥ 3 days, and excluding admission for diagnostic procedures).

Additional outcomes of interest included primary non-response (suboptimal response occurring ≤ 4 months of index date), secondary loss of response (suboptimal response occurring > 4 months after index date, among patients who did not experience primary non-response), time to treatment discontinuation, time to first surgery, and time to first hospitalization. Responses to the physician survey identified physician- and patient-related barriers to anti-TNF use.

Descriptive statistics included proportions for categorical variables and the mean ± standard deviation (SD) or median and range for continuous variables. The Kaplan–Meier method was used to assess the cumulative incidence of suboptimal response. Patients were censored at the end of the observation period or at treatment discontinuation if treatment discontinuation was unrelated to clinical response.

A *post-hoc* univariate and multivariate analysis using a Cox proportional model was applied to investigate baseline patient characteristics that were predictive of suboptimal response to anti-TNF therapy in patients with CD. All variables with a univariate p < 0.20 were included in the multivariate model. Age was a forced variable in the multivariate analysis. Where overlapping factors were identified in the univariate analysis, only the most relevant parameter was included.

Furthermore, a *post-hoc* univariate and multivariate logistic regression analysis was also conducted to identify potential predictors of primary non-response and secondary loss of response in patients with CD. Variables with a univariate p < 0.20 were included in the multivariate model; a limit of 1 covariate per 10 events was applied (those covariates with the highest significance were included). Age and sex were forced variables in the multivariate analyses.

Given the small number of patients with UC (and thus, event rate), *post-hoc* univariate and multivariate analyses were not conducted for the UC cohort.

## Results

### Demographic and clinical characteristics at index date

The EXPLORE study included 287 patients with IBD (UC, n = 35; CD, n = 252) treated with first-line anti-TNF therapy at 10 centers in mainland China. The majority of patients were male (72.1%) and the mean (SD) age of patients was 43.1 (14.2) years in the UC cohort and 31.9 (11.3) years in the CD cohort (Table 1). The median duration of IBD symptoms was 3.0 years (interquartile range 1.0, 6.0) for both patients with UC or CD. Among those for whom clinical disease activity was assessed, 100% (26/26) of patients with UC and 60.4% (58/96) of patients with CD had moderate or severe disease activity at the index date. The majority of patients with UC had extensive disease (60.0%, 21/35), while most patients with CD had ileocolonic disease (54.0%, 136/252). CD disease behavior was most often non-stricturing and non-penetrating (43.3%, 109/252), while 46.4% (117/252) of patients had perianal disease. A total of 77.1% (27/35) of patients with UC and 40.1% (101/252) of patients with CD received prior non-biologic therapy, with aminosalicylates being the most prescribed therapy in both groups. At the index date, 68.6% (24/35) of patients with UC and 42.9% (108/252) of patients with CD were receiving concomitant non-biologic therapy, most commonly aminosalicylates. The median (range) observational period was 27.6 (24, 60) months and 40.0 (24, 60) months for the UC and CD patient cohorts, respectively.

**Table 1:** Demographic and clinical characteristics of the study patients

	UC (n = 35)	CD (n = 252)
Observational period, months, median (min, max)	27.6 (24, 60)	40.0 (24, 60)
Male, n (%)	19 (54.3)	188 (74.6)
Age, years, mean (SD)	43.1 (14.2)	31.9 (11.3)
BMI, mean (SD)	19.48 (2.47)	18.90 (2.98)
Extraintestinal manifestations within 2 years, n (%)	4 (11.4)	4 (1.6)
*Duration of IBD, years, median (IQR)*		
Since appearance of IBD symptoms	3.0 (1.0, 6.0)	3.0 (1.0, 6.0)
Since diagnosis	1.0 (0.0, 4.0)	0.0 (0.0, 1.0)
IBD-related surgery since diagnosis, n (%)^a^	0 (0)	65 (26.6)
*UC disease location at diagnosis, n (%)*		
Proctitis involvement limited to the rectum	2 (5.7)	–
Left-sided involvement limited to the proportion of the colon distal to the splenic flexure	7 (20.0)	–
Extensive involvement extends proximal to the splenic flexure, including pancolitis	21 (60.0)	–
Unknown	5 (14.3)	–
*CD disease location at diagnosis, n (%)*^*b*^		
Ileal with upper GI disease (L1 + L4)	–	21 (8.3)
Ileal without upper GI disease (L1)	–	18 (7.1)
Colonic with upper GI disease (L2 + L4)	–	19 (7.5)
Colonic without upper GI disease (L2)	–	30 (11.9)
Ileocolonic with upper GI disease (L3 + L4)	–	32 (12.7)
Ileocolonic without upper GI disease (L3)	–	104 (41.3)
Unknown	–	28 (11.1)
*Disease activity, n (%)*		
Normal	0 (0.0)	13 (5.2)
Mild	0 (0.0)	25 (9.9)
Moderate	4 (11.4)	34 (13.5)
Severe	22 (62.9)	24 (9.5)
Unknown	9 (25.7)	156 (61.9)
*Biochemical activity, n (%)*		
Normal	5 (14.3)	52 (20.6)
Active	26 (74.3)	159 (63.1)
Unknown	4 (11.4)	41 (16.3)
*Disease behavior, n (%)*^*b*^		
Non-stricturing, non-penetrating with perianal disease (B1p)	–	60 (23.8)
Non-stricturing, non-penetrating without perianal disease (B1)	–	49 (19.4)
Stricturing with perianal disease (B2p)	–	40 (15.9)
Stricturing without perianal disease (B2)	–	30 (11.9)
Penetrating with perianal disease (B3p)	–	17 (6.7)
Penetrating without perianal disease (B3)	–	23 (9.1)
Unknown	–	33 (13.1)
*Prior non-biologic therapy, n (%)*		
Yes	27 (77.1)	101 (40.1)
No	7 (20.0)	147 (58.3)
Unknown	1 (2.9)	4 (1.6)
*Prior non-biologic therapy, n (%)*		
Aminosalicylates	21 (77.8)	66 (65.3)
Antibiotics	9 (33.3)	20 (19.8)
Corticosteroids	16 (59.3)	47 (46.5)
*Immunosuppressants*	11 (40.7)	47 (46.5)
Azathioprine	6 (22.2)	44 (43.6)
Mercaptopurine	3 (11.1)	0 (0.0)
Methotrexate	1 (3.7)	5 (5.0)
Thalidomide	0 (0.0)	6 (5.9)
Tacrolimus	1 (3.7)	1 (1.0)
Cyclosporine A	0 (0.0)	1 (1.0)
Nutritional therapies	2 (7.4)	33 (32.7)
Other	15 (55.6)	46 (45.5)
*Corticosteroid status*		
Intolerant	0	6 (2.4)
Dependent	13 (37.1)	23 (9.1)
Not dependent or intolerant	9 (25.7)	21 (8.3)
Unknown	13 (37.1)	202 (80.2)
Duration of non-biologic therapy discontinued before index date, months, mean (SD)^c^	1.2 (2.1)	1.1 (2.5)
Concomitant non-biologic therapy at index date, n (%)	24 (68.6)	108 (42.9)
Aminosalicylates	19 (54.3)	77 (30.6)
Corticosteroids	8 (22.9)	35 (13.9)
Immunosuppressants	5 (14.3)	22 (8.7)

*BMI* body mass index, *CD* Crohn’s disease, *GI* gastrointestinal, *IBD* inflammatory bowel disease, *IQR* interquartile range, *UC* ulcerative colitis, *SD* standard deviation

^a^IBD-related surgeries including total proctocolectomy, total and partial colectomy, ileocolonic bowel resection, small bowel resection, strictureplasty, perianal surgery, ileostomy reversal; ^b^Montreal classification; ^c^n = 31 for UC and n = 67 for CD

Demographic and clinical characteristics at index date were also categorized according to suboptimal response to first-line anti-TNF therapy (Additional file 1: Table S1).

### Biologic treatment patterns at index date

All patients with UC and CD received infliximab as their first-line anti-TNF therapy with a median (interquartile range) duration of treatment of 5.8 (1.4, 8.8) and 12.8 (7.6, 33.9) months, respectively (Table 2). Subsequent anti-TNF therapy with adalimumab was administered to 2.9% (1/35) of patients in the UC cohort and 0.4% (1/252) of patients in the CD cohort. A further 3.6% (9/252) of patients in the CD cohort reinitiated infliximab with a median (interquartile range) duration of treatment of 11.2 (7.1, 17.6) months.

**Table 2:** Anti-TNF treatment history

	UC (n = 35)	CD (n = 252)
First-line anti-TNF therapy, n (%)		
Infliximab	35 (100)	252 (100)
Treatment period, months, median (IQR)	5.8 (1.4, 8.8)	12.8 (7.6, 33.9)
Second anti-TNF therapy, n (%)	1 (2.9)	10 (4.0)
Adalimumab, n/N (%)	1/1 (100)	1/10 (10)
Treatment period, months, median	3.9 (3.9, 3.9)	5.0 (5.0, 5.0)
(IQR)		
Infliximab, n/N (%)	0	9/10 (90)
Treatment period, months, median (IQR)	–	11.2 (7.1, 17.6)

*CD* Crohn’s disease, *IQR* interquartile range, *TNF* tumor necrosis factor, *UC* ulcerative colitis

### Incidence of suboptimal response

**Fig. 1:**
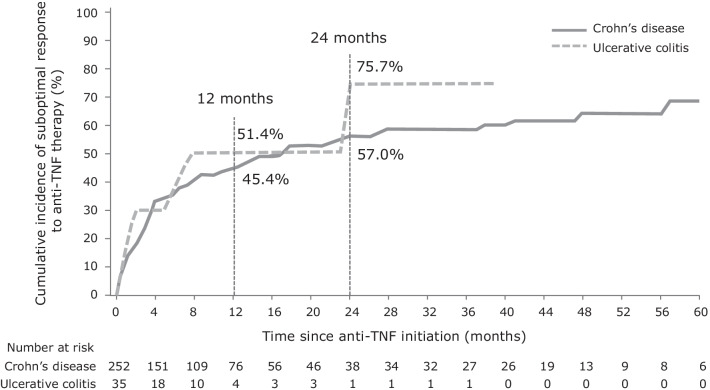
Cumulative incidence of suboptimal response to first-line anti-TNF therapy in patients with IBD in China. *IBD* inflammatory bowel disease, *TNF* tumor necrosis factor

In total, 45.7% (16/35) and 48.8% (123/252) of patients in the UC and CD cohorts, respectively, experienced a suboptimal response to their first-line anti-TNF therapy during the observation period. At 12 and 24 months, the cumulative incidence of suboptimal response to first-line anti-TNF treatment was 51.4% and 75.7% in patients with UC, and 45.4% and 57.0% in patients with CD, respectively (Fig. 1). The median time to the first suboptimal response was 7.2 months in patients with UC and 14.3 months in patients with CD.

### Indicators of suboptimal response

**Fig. 2:**
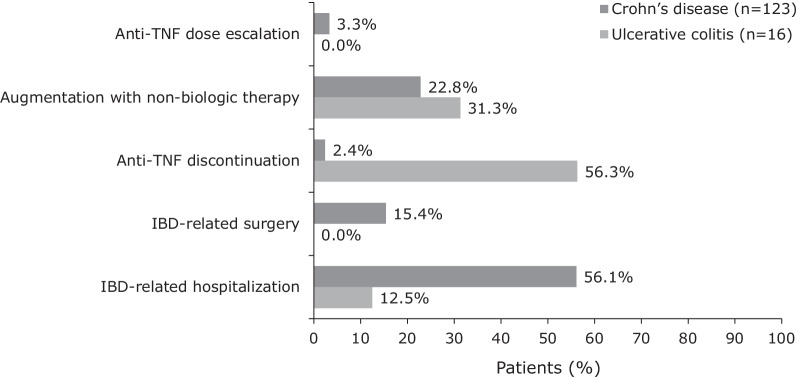
First indicators of suboptimal response to first-line anti-TNF therapy in patients with IBD in China. *IBD* inflammatory bowel disease, *TNF* tumor necrosis factor

For patients with UC and a suboptimal response to anti-TNF therapy, the most frequent first indicator was discontinuation of anti-TNF therapy (9/16, 56.3%), while the most frequent first indicator for patients with CD was IBD-related hospitalization (69/123, 56.1%). Augmentation with non-biologic therapy was the second most common first indicator of a suboptimal response in both UC (5/16, 31.3%) and CD (28/123, 22.8%) cohorts (Fig. 2). Dose escalation was the least frequent first indicator of suboptimal response to anti-TNF therapy (0.0% for UC and 3.3% for CD).

**Fig. 3:**
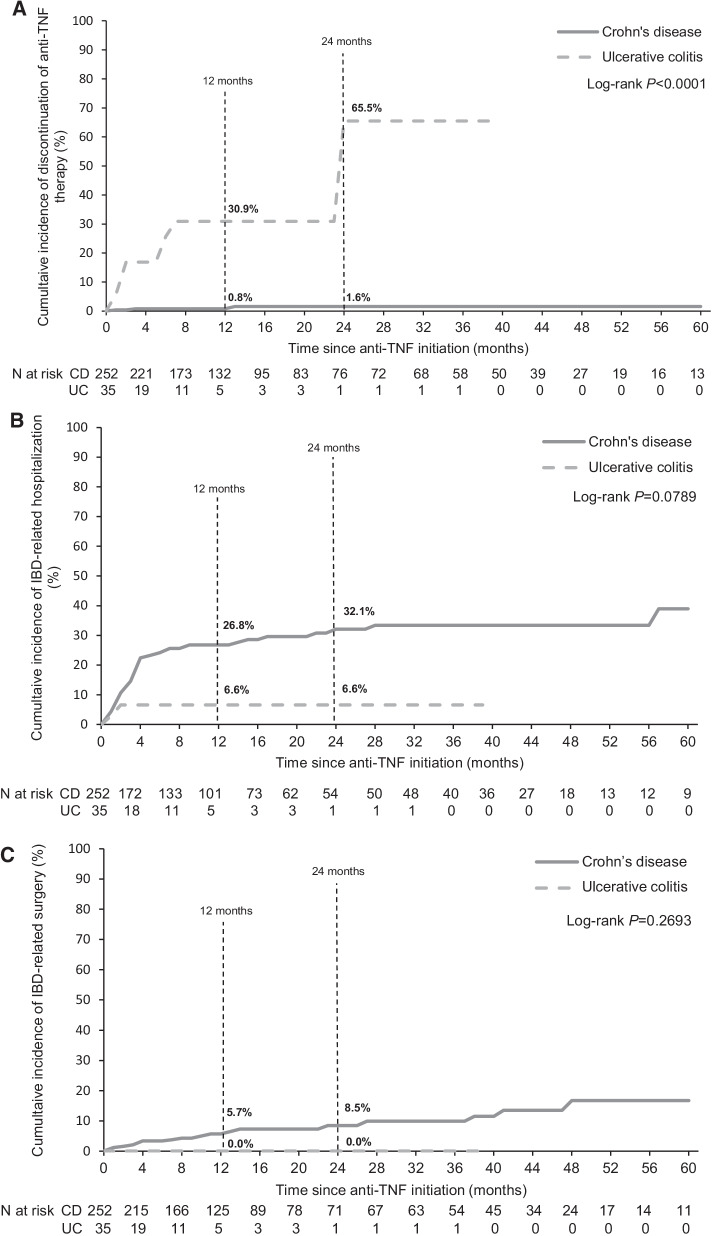
Cumulative incidence of **A** discontinuation of anti-TNF therapy, **B** IBD-related hospitalizations and **C** surgery as indicators of suboptimal response in patients with IBD in China. *CD* Crohn’s disease, *IBD* inflammatory bowel disease, *TNF* tumor necrosis factor, *UC* ulcerative colitis

The cumulative incidence of discontinuation of first-line anti-TNF therapy was 30.9% and 65.5% for patients with UC at 12 and 24 months, respectively, compared with 0.8% and 1.6% of patients with CD (Fig. 3A). For IBD-related hospitalizations, the cumulative incidence of this event at 12 and 24 months was 6.6% for UC at both timepoints, and 26.8% and 32.1% for CD, respectively (Fig. 3B). No patients with UC in China experienced an IBD-related surgery during the initial 24 months of anti-TNF initiation, whereas the cumulative incidence of a surgical intervention was 5.7% and 8.5% in patients with CD, at 12 and 24 months, respectively (Fig. 3C).

### Primary non-response and secondary loss of response

**Fig. 4:**
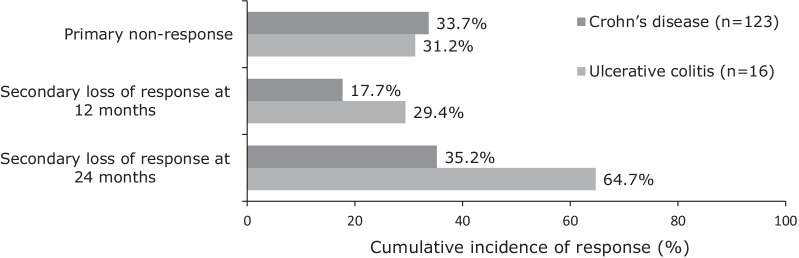
Cumulative incidence of primary non-response and secondary loss of response to anti-TNF therapy in patients with IBD in China. *IBD* inflammatory bowel disease, *TNF* tumor necrosis factor

The cumulative incidence of primary non-response was 31.2% and 33.7% in the UC and CD patient cohorts during the observation period, respectively (Fig. 4). The cumulative incidence of secondary loss of response (in those patients not experiencing primary loss of response) at 12 and 24 months was 29.4% and 64.7% in patients with UC, respectively, and 17.7% and 35.2% in patients with CD.

### Predictors of response to first-line anti-TNF therapy in patients with CD

**Fig. 5:**
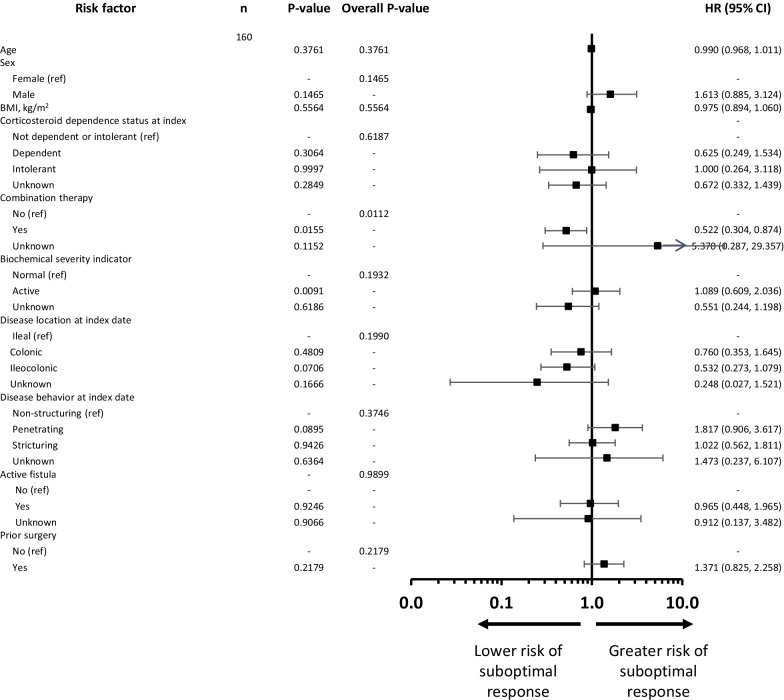
Multivariate Cox proportional hazards model for potential predictors of suboptimal response to anti-TNF therapy over time in patients with CD in China. *BMI*, body mass index, *CD* Crohn’s disease, *CI* confidence interval, *HR* hazard ratio, *TNF* tumor necrosis factor, *Ref* Reference risk factor level. P-value, Test assessing if there is any difference in the event rate for this level of the risk factor versus the reference level. Overall P-value, Test assessing if there is any difference in the event rate across the different levels of the risk factor

Univariate analyses indicated that anti-TNF plus immunosuppressant combination therapy, biochemical severity index, disease behavior at index date, and prior surgery were predictive of suboptimal response in patients with CD (Additional file 1: Fig. S1). Patient sex, body mass index, corticosteroid dependence status at baseline, use of immunosuppressant therapy at index date, disease location at diagnosis, and the presence of an active fistula were also included as variables in the multivariate analysis. Following the multivariate analysis, only the use of combination therapy was protective against sub-optimal response to anti-TNF therapy, reducing the risk of sub-optimal response by 48% (Fig. 5).

An additional analysis was also conducted in patients with CD to establish predictors for primary non-response and secondary loss of response (univariate analysis not reported). No risk factors were identified for primary non-response (Additional file 1: Fig. S2) while prior surgery at baseline doubled the risk of secondary loss of response (Additional file 1: Fig. S3).

### Physician survey

A total of 10 participating physicians who had a mean (SD) 15.6 (7.6) years’ experience treating IBD responded to the physician survey. The mean (SD) length of experience using anti-TNF agents was 7.7 (4.9) years. A mean (SD) of 232.3 (183.4) biologic-naïve patients with UC and 216.3 (289.6) with CD were referred to an IBD specialist center during 2016. A mean (SD) proportion of 18.2% (27.4%) of patients with UC and 45.0% (22.4%) of patients with CD who were medically indicated for treatment with anti-TNF therapy were estimated not to have received it.

All participating physicians cited at least three barriers to prescribing anti-TNF therapies to patients with IBD. Patient-related barriers to prescribing were reported by all physicians, most commonly financial barriers (9/10, 90.0%), fear of side effects (5/10, 50.0%) and patient age (4/10, 40.0%). Nine (90.0%) physicians also reported physician-related barriers, including a perceived safety risk (6/10, 60.0%) and surgical treatment being used prior to anti-TNF prescribing (3/10, 30.0%). Reimbursement was also considered to be a barrier to prescribing anti-TNF therapies by seven (70.0%) physicians.

## Discussion

To our knowledge, the EXPLORE study is the first to comprehensively describe the suboptimal responses among patients with IBD treated with anti-TNF therapy in real-world clinical practice in China. We found that more than 1 in 2 patients with IBD in China were at risk of experiencing a suboptimal response to first-line anti-TNF therapy at 2 years post-initiation. The most frequently reported sub-optimal response indicators were anti-TNF discontinuation (for UC), IBD-related hospitalization (for CD) and augmentation with non-biologic therapy (for both UC and CD). Although conducted in only a small number of physicians, our survey also found that IBD specialists in mainland China remain skeptical about the use of anti-TNF therapy due to concerns about associated safety risks and immunogenicity. Together, our study highlights the substantial unmet medical need and suboptimal treatment outcomes with anti-TNF agents for the treatment of IBD in China.

The interpretation of data from the present study should take into consideration the fact that, at the time of this study, infliximab was the only approved anti-TNF for treating CD in China and marketing approval had not been granted for the use of infliximab in UC. As such, relatively few patients with UC were included in this study, the majority of whom had severe disease. Disease severity could be one explanation for the high suboptimal response rate to first-line anti-TNF therapy in the UC cohort. Our data among patients with CD may better reflect the real-world clinical pattern of patients with IBD in China. Within the CD cohort, IBD-related hospitalization was the most frequently reported first indicator of suboptimal response, reported in approximately one-third of patients receiving anti-TNF therapy. It is of concern that no prior indicator of suboptimal response, such as dose escalation, anti-TNF discontinuation, or augmentation with non-biologic therapy, was reported in these patients, as hospitalization is associated with substantial economic burden and may expose patients to nosocomial infections [15, 16]. It also highlights the suboptimal effectiveness of anti-TNF therapies in a substantial proportion of patients with IBD, which was further confirmed in our finding that 1 in 3 patients with CD experienced primary non-response to anti-TNF therapy. Interestingly, the anti-TNF discontinuation rate in patients with CD is low despite high rates of IBD-related hospitalization and primary non-response, potentially due to the lack of alternative therapies. In contrast, the anti-TNF discontinuation rate was high among patients with UC, possibly related to the fact that anti-TNF therapies were used in an off-label manner at that time. Together, our data highlight the suboptimal effectiveness of anti-TNF therapies as used in China in IBD as well as the lack of treatment options beyond anti-TNF therapies, at the time the study was conducted.

Augmentation with non-biologic therapy was also frequently reported as an indicator of suboptimal response in patients with CD. Concomitant immunosuppressive therapy is recommended in patients with CD receiving infliximab to reduce the risk of immunogenicity [17]. Our multivariate analysis of patients with CD in this subgroup analysis indicated that combining anti-TNF treatment with immunosuppressant therapy reduced the risk of a suboptimal response. However, combination use of anti-TNF therapy and thiopurines is associated with increased risk of malignancies including skin cancer and lymphoma [18–21]. This association may contribute to the perceived safety risks of anti-TNF therapies among physicians, as reported in our physician survey.

The cumulative incidences of suboptimal response observed were numerically higher than those observed in the overall EXPLORE population (2 years: UC, 76% vs 33%; CD, 57% vs 41%) [5], but are largely consistent with other real-world studies [7, 9]. A retrospective, multicenter chart review study in Europe and Canada found 64% of patients with UC and 58% of patients with CD were at risk of experiencing suboptimal therapy at 2 years of initiating anti-TNF treatment [7]. A separate US study reported a suboptimal response rate of 86% after 2 years of anti-TNF therapy [9]. Median times to first suboptimal response in this analysis (UC: 7.2 months, CD: 14.3 months) were substantially shorter than those in the multinational chart review in Europe and Canada (UC: 12.5 months, CD: 17.5 months) [7] and were characterized by a high proportion of patients (> 30%) having a primary non-response to anti-TNF treatment. The differences in the level of and time to suboptimal responses reported in China versus other regions is multifactorial but may be attributed to differences in prescribing patterns for anti-TNF therapy. In particular, affordability was frequently cited as a perceived barrier to biologic prescribing for patients with IBD in China. At the time the survey was undertaken, infliximab was not reimbursable for the treatment of IBD in China. Accordingly, a typical patient with IBD treated with anti-TNF therapy in China is likely to have been treated with a biologic therapy relatively later than patients in Western countries.

Real-world studies in Western IBD populations have reported dose escalation and discontinuation of therapy as the most common indicators of a suboptimal response [7, 9]. Notably, dose escalation was rare in China (0–3.3%) compared with the US (30–34% of patients within 2 years) and the European and Canadian study (21–30%), likely due to cost barriers favoring a switch to non-biologic immunomodulators. Furthermore, patients in Europe and Canada with CD were more likely to have been prescribed a non-biologic therapy at the index date (70.6%) compared with the population in mainland China investigated in this study (42.9%), potentially limiting the use of augmentation as a treatment strategy in earlier studies [7]. Notably, the augmentation approach was favored over dose escalation in this subgroup analysis, despite Asian populations also having a high prevalence of *NUDT15* polymorphisms (10–20%) that are associated with thiopurine-related leukopenia [22]. In addition, the absence of routine therapeutic drug monitoring for patients with IBD in China at the time this study was undertaken, alongside cost barriers and infusion capacity, was likely a major limiting factor in the use of dose escalation as a treatment strategy to overcome suboptimal responses. However, the proportion of patients with CD in China requiring IBD-related hospitalization (32.1%) after 2 years was comparable with the overall post-1990 10-year rate for surgery among patients with CD reported in a systematic review and meta-analysis (38.7%) [23].

Several limitations need to be considered when interpreting the results of this post-hoc analysis. The nature of retrospective chart review studies means that suboptimal response indicators may be underestimated due to insufficient or inconsistent information being recorded in patient medical records and the reasoning for clinicians adjusting treatment regimen and patient outcomes (biochemical, clinical, endoscopic or quality of life) have not been captured. The off-label use of anti-TNF therapy and low numbers of patients with UC mean that the data set analyzed here must be interpreted with caution. While only 10 centers in mainland China participated in this study, potentially limiting the generalizability of the results, centers were located in North, East and South China, and included some of the largest anti-TNF prescribing centers in the country. Nonetheless, this study provides insights on the outcomes following first-line treatment with an anti-TNF therapy for IBD in China; the definition of suboptimal outcomes in this study was well established and aligned with a previous multinational chart review, allowing meaningful comparisons between countries [7, 9].

## Conclusions

To the authors’ knowledge, these data are the first to demonstrate that over half of the patients with IBD in China who were prescribed anti-TNF therapy as their first-line biologic therapy are at risk of experiencing suboptimal response at 2 years of anti-TNF initiation. The most common indicators of suboptimal response were IBD-related hospitalization and anti-TNF discontinuation. We also found that approximately 1 in 3 patients with IBD experienced primary non-response. With the evidence for high risk of suboptimal response and primary non-response, the need for biologics with new mechanisms of action as alternative treatment options to anti-TNF agents is warranted in China. Our study adds to the current evidence base on the unmet need associated with anti-TNF therapies in China, where earlier recognition of treatment failures to allow timely alternative treatment decisions, revised reimbursement policies and/or new therapeutic options may improve long-term outcomes in patients with IBD.

## Supplementary Information

**Additional file 1:** Physician survey, patient demographic and clinical characteristics. Full results of univariate analysis and multivariate logistic regression analysis.

## Data Availability

The datasets, including the redacted study protocol, redacted statistical analysis plan, and individual patient data supporting the results reported in this article, will be made available within 3 months from initial requests, to researchers who provide a methodologically sound proposal. The data will be provided after its de-identification, in compliance with applicable privacy laws, data protection and requirements for consent and anonymization. Proposals should be directed to the corresponding author. Data requestors will need to sign a data access agreement.

## Abbreviations

BMI: Body mass index
CD: Crohn’s disease
CI: Confidence interval
GI: Gastrointestinal
HR: Hazard ratio
IBD: Inflammatory bowel disease
IQR: Interquartile range
PNR: Primary non-response
SD: Standard deviation
SLOR: Secondary loss of response
TNF: Tumor necrosis factor
UC: Ulcerative colitis
