# Supplementary material for: Indicators of suboptimal response to anti-tumor necrosis factor therapy in patients from China with inflammatory bowel disease: results from the EXPLORE study
Source: BMC Gastroenterol. 2022 Feb 4;22:44. doi: 10.1186/s12876-021-02074-z (PMC8817491; doi:10.1186/s12876-021-02074-z)
Supplement: Supplementary file 1 — Additional file 1: Physician survey, patient demographic and clinical characteristics. Full results of univariate analysis and multivariate logistic regression analysis. [file 12876_2021_2074_MOESM1_ESM.docx]

**Indicators of suboptimal response to anti-tumor necrosis factor therapy in patients from China with inflammatory bowel disease: Results from the EXPLORE study**

Ji Li; Zhanju Liu; Pinjin Hu; Zhonghui Wen; Qian Cao; Xiaoping Zou; Yan Chen; Yingde Wang; Jie Zhong; Xizhong Shen; Dirk Demuth; Olga Fadeeva; Li Xie; Jun Chen; Jiaming Qian

**List of Supplementary Materials**

Supplementary Appendix 1. EXPLORE: IBD-Experienced Physician Survey

**Supplementary Table 1.** Demographic and clinical characteristics of study patients according to presence or absence of suboptimal response to first-line anti-TNF therapy

**Supplementary Figure 1.** Univariate analysis of potential predictors of suboptimal response to first-line anti-TNF therapy over time in patients with CD in China

**Supplementary Figure 2.** Multivariate logistic regression analysis for potential predictors of primary non-response to first-line anti-TNF therapy in patients with CD in China

**Supplementary Figure 3.** Multivariate logistic regression analysis for potential predictors of secondary loss of response to first-line anti-TNF therapy in patients with CD in China

Supplementary Appendix 1. EXPLORE: IBD-Experienced Physician Survey

1. How many years of experience do you have in treating IBD patients? ………..years
2. How many years of experience do you have in using anti-TNF therapy in IBD patients? ……….years
3. What is the estimated proportion of IBD patients managed in your gastroenterology department, out of the total number of gastroenterology patients managed in your department during 2016?

UC …..%

CD …..%

1. What was the estimated number of biologic-naive ulcerative colitis (UC) patients (adults) being referred to your center during 2016?

………. patients

1. What was the estimated number of biologic-naive Crohn’s disease (CD) patients (adults) being referred to your center during 2016?

………………patients

1. Approximatley, how many UC/CD patients (adults) referred to your centre in 2016 (%) do you think were **medically indicated** for treatment with anti-TNF therapy but **did not actually receive it**?

UC: ………………%

CD: ………………%

***We would like to understand the barriers you face, as an IBD-experienced physician, in prescribing anti-TNF therapy in your centre.***

1. Why do some of your IBD patients who are medically indicated for anti-TNF therapy, not receive such treatment? *(Please tick all that apply)*:

- No barriers to prescribing anti-TNF therapy *(i.e. all eligible patients are able to receive anti-TNF therapy)*

Patient-related barriers

- Patient age
- Patient perceived lack of efficacy
- Patient fear of side effects
- Patient cannot afford anti-TNF therapy/lack of insurance reimbursement
- Patient lives too far from facilities (e.g.: specialist centre / hospital or infusion centres)
- Patient choice of not being on these therapies
- Other *(Please specify)*:____________________

Physician-related barriers

- Concern of poor compliance
- Perceived safety risk
- Late referral to IBD specialist care center
- Surgical intervention before anti-TNF treatment considered
- Absence of national guidelines
- Absence of local (formulary) guidelines
- Complex process to obtain reimbursement / funding of anti-TNF therapy
- Limited capacity to conduct anti-TNF infusions *(please specify further details – tick all that apply)*:
  - Not enough infusion centres
  - Not enough infusion chairs within infusion centres
  - Shared infusion facilities with other patients (e.g. rheumatiod arthritis, psoriasis) requiring anti-TNF therapy
  - Not enough staff support (e.g. nurse) within infusion centres to monitor patients during the infusion
  - Other *(Please specify)*: _____________
- Other *(Please specify)*:____________________

Reimbursement-related

- Budget restrictions at the centre
- Require specific/complex process for reimbursement of anti-TNF therapy
- Other reimbursement restrictons *(Please specify)*: ___________________________________

*Of those response options ticked, please identify the* ***Top*** *3 reasons, starting with the most important reason.*

1)____________

2)____________

3)____________

***We would now like to understand, more broadly, what you think are the barriers faced by general gastroenterologists in your country (i.e. non IBD-experienced gastroenterologist), when prescribing anti-TNF therapy for IBD patients.***

1. Why do IBD patients who are medically indicated for anti-TNF therapy, not receive such treatment? *(Please tick all that apply)*

- No barriers to prescribing anti-TNF therapy *(i.e. all eligible patients are able to receive anti-TNF therapy)*

Patient-related

- Patient age
- Patient perceived lack of efficacy
- Patient fear of side effects
- Patient cannot afford anti-TNF therapy/lack of insurance reimbursement
- Patient lives too far from facilities (e.g.: specialist centre/hospital or infusion centres)
- Patient choice of not being on these therapies
- Other *(Please specify)*:____________________

Physician-related

- Lack of experience with anti-TNF therapy
- Perceived lack of efficacy
- Perceived safety risk
- Perceived efficacy of anti-TNF therapy is comparable to other therapies such as immunomodulator, corticosteroids, etc.
- Perception that only specialist IBD centres/tertiray centres are allowed to prescribe anti-TNF therapy
- Absence of national guidelines
- Absence of local (formulary) guidelines
- Non-availability of anti-TNF therapy at their site
- Late diagnosis of IBD
- Late referral by primary care physician
- Surgical intervention before anti-TNF treatment considered
- Limited capacity to conduct anti-TNF infusions *(Please specify further details – tick all that apply):*
  - Not enough infusion centres
  - Not enough infusion chairs within infusion centres
  - Shared infusion facilities with other patients (e.g. rheumatiod arthritis, psoriasis) requiring anti-TNF therapy
  - Not enough staff support (e.g. nurse) within infusion centres to monitor patients during the infusion
  - Other *(Please specify)*: _____________
- Other *(Please specify)*:____________________

Reimbursement-related

- Budget restrictions at the centre
- Require specific/complex process for reimbursement of anti-TNF therapy
- Other reimbursement restrictons *(Please specify)*: ___________________________________

*Of those response options ticked, please identify the* ***Top*** *3 reasons, starting with the most important reason.*

1)____________

2)____________

3)____________

***We would now like to understand how patients who are prescribed anti-TNF therapies at your centre are receiving their infusions/injections.***

1. Where do your IBD patients go to receive their anti-TNF infusion/injection? *(Please provide the patient distribution to your best estimate)*
2. Infusion/injection at your centre/hospital ……..%
3. Satellite infusion centre (community centre) ……..%
4. Home infusion ……..%
5. Self-injection at home ……..%
6. Others *(Please specify)*:

1.________, ……..%

2.________, ……..%

1. Generally, how long does it take your patients to travel to receive their anti-TNF infusion/injection? *(Please provide the patient distribution to your best estimate)*
2. No travel required ........%
3. Less than 1 hour ……..%
4. 1 to 2 hours ……..%
5. More than 2 hours ……..%

11.a. On average, how long does it take to complete each intravenous infusion of anti-TNF (including monitoring time after the infusion has been completed)?

______hours ______minutes

______Not applicable

- 1. In terms of the infusion centres **your patients** are visiting, what do you think are the main challenges for them in receiving their infusion, once they are prescribed an anti-TNF therapy?
- No challenges
- Not enough infusion centres
- Infusion centres too far for patienst to travel to
- Not enough infusion chairs within the infusion centres
- Shared infusion facilities with other patients (e.g. rheumatiod arthritis, psoriasis) requiring anti-TNF therapy
- Not enough staff support (e.g. nurse) within infusion centres to conduct the infusion/monitor patients
- Other *(Please specify)*: _____________

*Of those response options ticked, please identify the* ***Top 3*** *challenges, starting with the most important one.*

1)____________

2)____________

3)____________

**Supplementary Table 1.** Demographic and clinical characteristics of study patients according to presence or absence of suboptimal response to first-line anti-TNF therapy

|  | **UC patients** | | | **CD patients** | | |
| --- | --- | --- | --- | --- | --- | --- |
|  | **Overall**  **(n=35)** | **Suboptimal response to first anti-TNF therapy (N=16)** | **No documented suboptimal response**  **(N=19)** | **Overall (n=252)** | **Suboptimal response to first anti-TNF therapy (N=123)** | **No documented suboptimal response**  **(N=129)** |
| **Observational period, months, median (min, max)** | 27.6 (24, 60) | 37.8 (24,60) | 24.1 (24,58) | 40.0 (24, 60) | 39.9 (24,60) | 40.2 (24,60) |
| **Male, n (%)** | 19 (54.3) | 6 (37.5) | 13 (68.4) | 188 (74.6) | 96 (78.1) | 92 (71.3) |
| **Age, years, mean (SD)** | 43.1 (14.2) | 44.2 (17.5) | 42.2 (11.2) | 31.9 (11.3) | 31.1 (10.5) | 32.5 (12.1) |
| **BMI, mean (SD)** | 19.48 (2.47) | 18.57 (2.20) | 21.06 (2.19) | 18.90 (2.98) | 18.64 (3.22) | 19.17 (2.70) |
| **Extraintestinal manifestations within 2 years, n (%)** | 4 (11.4) | 3 (18.8) | 1 (5.3) | 4 (1.6) | 3 (2.4) | 1 (0.8) |
| **Duration of IBD, years, median (IQR)**  Since appearance of IBD symptoms  Since diagnosis | 3.0 (1.0, 6.0)  1.0 (0.0, 4.0) | 2.5 (1.0,4.0)  1.5 (0.0,3.5) | 3.0 (1.0,6.0)  1.0 (0.0,5.0) | 3.0 (1.0, 6.0)  0.0 (0.0, 1.0) | 3.0 (1.0,6.0)  0.0 (0.0,1.0) | 3.0 (1.0,6.0)  0.0 (0.0,1.5) |
| **IBD-related surgery since diagnosis, n (%)^a^** | 0 (0) | 0 (0) | 0 (0) | 65 (26.6) | 42 (34.0) | 23 (17.8) |
| **UC disease location at diagnosis, n (%)**  Proctitis involvement limited to the rectum  Left-sided involvement limited to the proportion of the colon distal to the splenic flexure  Extensive involvement extends proximal to the splenic flexure, including pancolitis  Unknown | 2 (5.7)  7 (20.0)  21 (60.0)  5 (14.3) | 1 (6.3)  3 (18.8)  10 (62.5)  2 (12.5) | 1 (5.3)  4 (21.1)  11 (57.9)  3 (15.8) | –  –  –  – |  |  |
| **CD disease location at diagnosis, n (%)^b^**  Ileal with upper GI disease (L1 + L4)  Ileal without upper GI disease (L1)  Colonic with upper GI disease (L2 + L4)  Colonic without upper GI disease (L2)  Ileocolonic with upper GI disease (L3 + L4)  Ileocolonic without upper GI disease (L3)  Unknown | –  –  –  –  –  –  – |  |  | 21 (8.3)  18 (7.1)  19 (7.5)  30 (11.9)  32 (12.7)  104 (41.3)  28 (11.1) | 15 (12.2)  9 (7.3)  9 (7.3)  12 (9.8)  18 (14.6)  52 (42.3)  8 (6.5) | 6 (4.7)  9 (7.0)  10 (7.8)  18 (14.0)  14 (10.9)  52 (40.3)  20 (15.5) |
| **Disease activity, n (%)**  Normal  Mild  Moderate  Severe  Unknown | 0 (0.0)  0 (0.0)  4 (11.4)  22 (62.9)  9 (25.7) | 0 (0.0)  0 (0.0)  1 (6.3)  12 (75.0)  3 (18.7) | 0 (0.0)  0 (0.0)  3 (15.8)  10 (52.6)  6 (31.6) | 13 (5.2)  25 (9.9)  34 (13.5)  24 (9.5)  156 (61.9) | 6 (4.9)  15 (12.2)  16 (13.0)  10 (8.1)  76 (61.8) | 7 (5.4)  10 (7.8)  18 (14.0)  14 (10.9)  80 (62.0) |
| **Biochemical activity, n (%)**  Normal  Active  Unknown | 5 (14.3)  26 (74.3)  4 (11.4) | 3 (18.8)  11 (68.8)  2 (12.5) | 2 (10.5)  15 (79.0)  2 (10.5) | 52 (20.6)  159 (63.1)  41 (16.3) | 26 (21.1)  84 (68.3)  13 (10.6) | 26 (20.2)  75 (58.1)  28 (21.7) |
| **Disease behavior, n (%)^b^**  Non-stricturing, non-penetrating with perianal disease (B1p)  Non-stricturing, non-penetrating without perianal disease (B1)  Stricturing with perianal disease (B2p)  Stricturing without perianal disease (B2)  Penetrating with perianal disease (B3p)  Penetrating without perianal disease (B3)  Unknown | –  –  –  –  –  –  – |  |  | 60 (23.8)  49 (19.4)  40 (15.9)  30 (11.9)  17 (6.7)  23 (9.1)  33 (13.1) | 32 (26.0)  21 (17.1)  19 (15.5)  16 (13.0)  10 (8.1)  15 (12.2)  10 (8.1) | 28 (21.7)  28 (21.7)  21 (16.3)  14 (10.9)  7 (5.4)  8(6.2)  23 (17.8) |
| **Prior non-biologic therapy, n (%)**  Yes  No  Unknown | 27 (77.1)  7 (20.0)  1 (2.9) | 11 (68.8)  5 (31.3)  0 (0.0) | 16 (84.2)  2 (10.5)  1 (5.3) | 101 (40.1)  147 (58.3)  4 (1.6) | 43 (35.0)  78 (63.4)  2 (1.6) | 58 (45.0)  69 (53.5)  2 (1.6) |
| **Prior non-biologic therapy, n (%)**  Aminosalicylates  Antibiotics  Corticosteroids  Immunosuppressants  Azathioprine  Mercaptopurine  Methotrexate  Thalidomide  Tacrolimus  Cyclosporine A  Nutritional therapies  Other | 21 (77.8)  9 (33.3)  16 (59.3)  11 (40.7)  6 (22.2)  3 (11.1)  1 (3.7)  0 (0.0)  1 (3.7)  0 (0.0)  2 (7.4)  15 (55.6) | 8 (72.7)  2 (18.2)  8 (72.7)  5 (45.5)  4 (36.4)  0 (0.0)  0 (0.0)  0 (0.0)  1 (9.1)  0 (0.0)  1 (9.1)  3 (27.3) | 13 (81.3)  7 (43.8)  8 (50.0)  6 (37.5)  2 (12.5)  3 (18.8)  1 (6.3)  0 (0.0)  0 (0.0)  0 (0.0)  1 (6.3)  12 (75.0) | 66 (65.3)  20 (19.8)  47 (46.5)  47 (46.5)  44 (43.6)  0 (0.0)  5 (5.0)  6 (5.9)  1 (1.0)  1 (1.0)  33 (32.7)  46 (45.5) | 28 (65.1)  11 (25.6)  24 (55.8)  23 (53.5)  20 (46.5)  0 (0.0)  2 (4.7)  4 (9.3)  1 (2.3)  1 (2.3)  16 (37.2)  17 (39.5) | 38 (65.5)  9 (15.5)  23 (39.7)  24 (41.4)  24 (41.4)  0 (0.0)  3 (5.2)  2 (3.4)  0 (0.0)  0 (0.0)  17 (29.3)  29 (50.0) |
| **Corticosteroid status**  Intolerant  Dependent  Not dependent or intolerant  Unknown | 0  13 (37.1)  9 (25.7)  13 (37.1) | 0  9 (56.3)  5 (31.2)  2 (12.5) | 0  4 (21.1)  4 (21.1)  11 (57.9) | 6 (2.4)  23 (9.1)  21 (8.3)  202 (80.2) | 4 (3.3)  12 (9.8)  15 (12.2)  92 (74.8) | 2 (1.5)  11 (8.5)  6 (4.7)  110 (85.3) |
| **Duration of non-biologic therapy discontinued before index date, months, mean (SD)^c^** | 1.2 (2.1) | 2.1 (2.4) | 0.9 (2.0) | 1.1 (2.5) | 1.3 (3.0) | 0.8 (1.9) |
| **Concomitant non-biologic therapy at index date, n (%)** | 24 (68.6) | 10 (62.5) | 14 (87.5) | 108 (42.9) | 43 (34.1) | 65 (50.4) |
| Aminosalicylates | 19 (54.3) | 8 (50.0) | 11 (57.9) | 77 (30.6) | 31 (24.6) | 46 (35.7) |
| Corticosteroids | 8 (22.9) | 2 (12.5) | 6 (31.6) | 35 (13.9) | 15 (11.9) | 20 (15.5) |
| Immunosuppressants | 5 (14.3) | 2 (12.5) | 3 (15.8) | 22 (8.7) | 6 (4.8) | 16 (12.4) |

BMI, body mass index; CD, Crohn’s disease; GI, gastrointestinal; IBD, inflammatory bowel disease; IQR, interquartile range; UC, ulcerative colitis; SD, standard deviation

**Supplementary Figure 1.** Univariate analysis of potential predictors of suboptimal response to first-line anti-TNF therapy over time in patients with CD in China


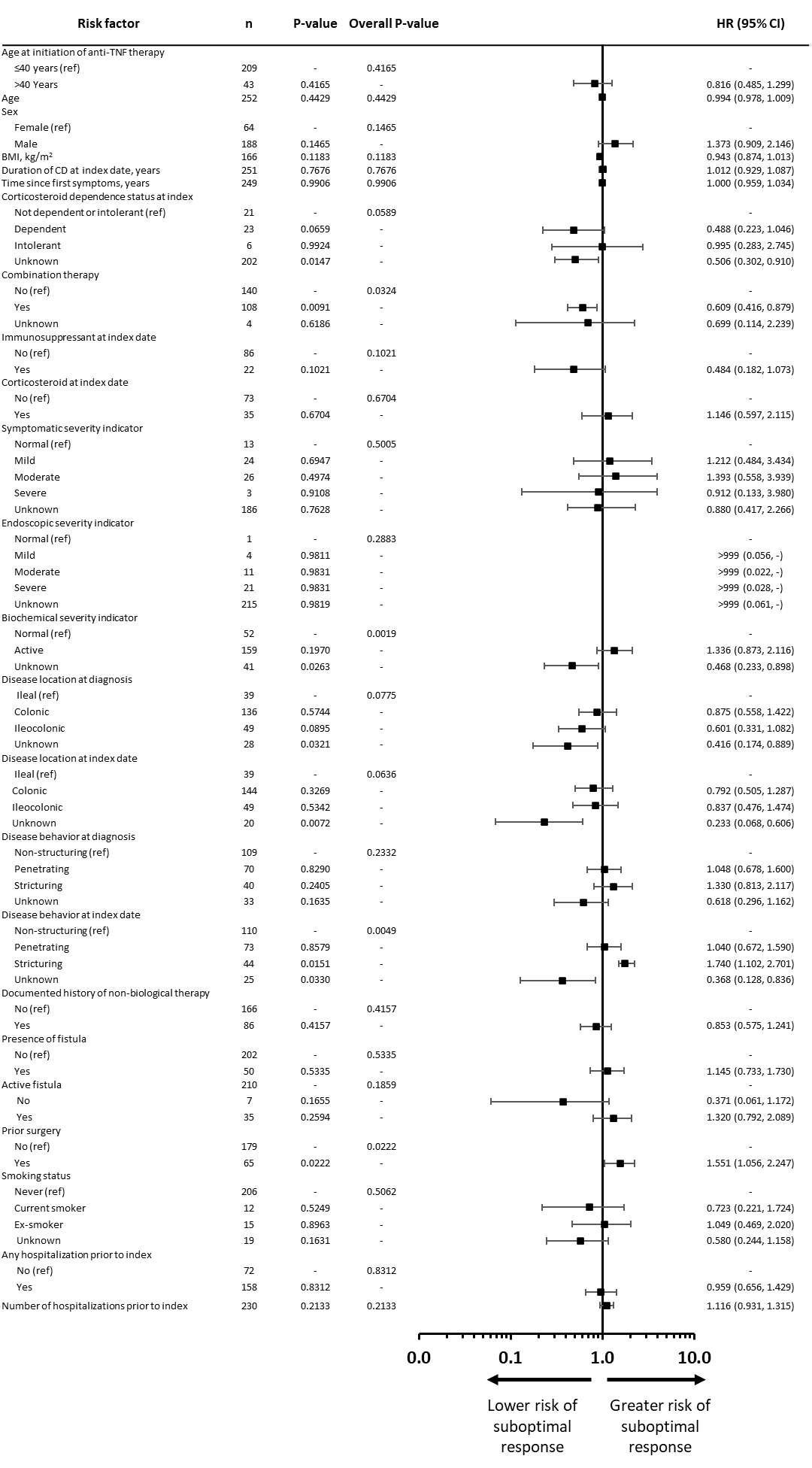


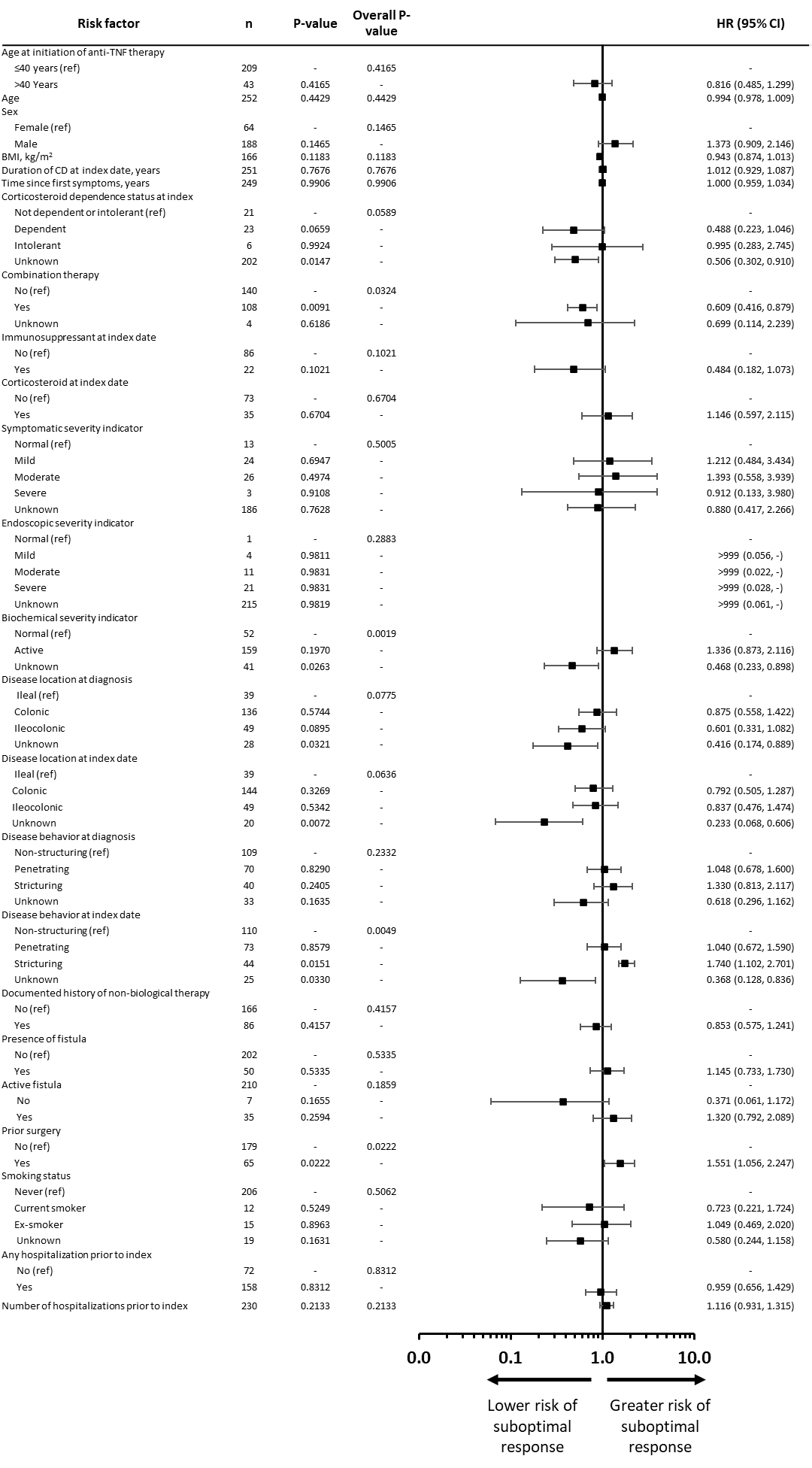


BMI, body mass index; CD, Crohn’s disease; CI, confidence interval; HR, hazard ratio; Ref, Reference risk factor level; TNF, tumor necrosis factor

P-value, Test assessing if there is any difference in the event rate for this level of the risk factor versus the reference level

Overall P-value, Test assessing if there is any difference in the event rate across the different levels of the risk factor

**Supplementary Figure 2.** Multivariate logistic regression analysis for potential predictors of primary non-response to first-line anti-TNF therapy in patients with CD in China

**
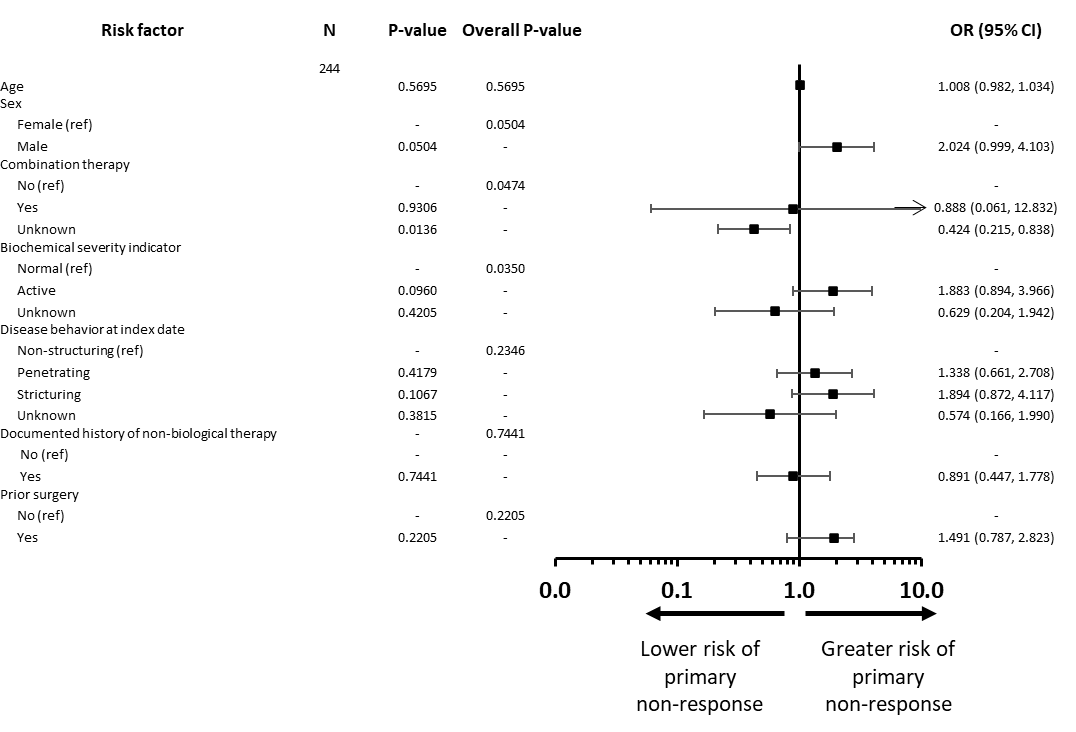
**

CD, Crohn’s disease; CI, confidence interval; OR, odds ratio; Ref, Reference risk factor level; TNF, tumor necrosis factor

P-value, Test assessing if there is any difference in the event rate for this level of the risk factor versus the reference level

Overall P-value, Test assessing if there is any difference in the event rate across the different levels of the risk factor

**Supplementary Figure 3.** Multivariate logistic regression analysis for potential predictors of secondary loss of response to first-line anti-TNF therapy in patients with CD in China


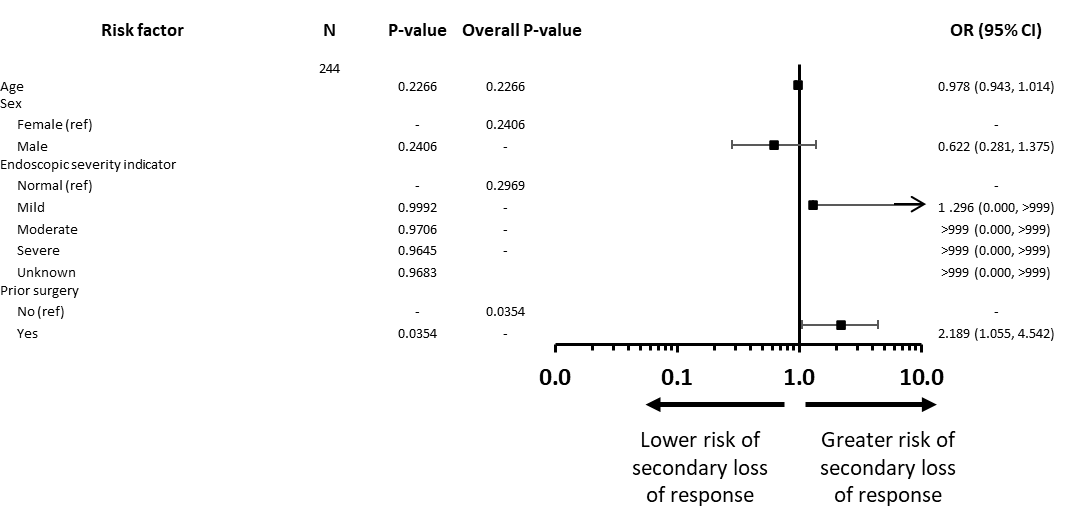


CD, Crohn’s disease; CI, confidence interval; OR, odds ratio; Ref, Reference risk factor level; TNF, tumor necrosis factor

P-value, Test assessing if there is any difference in the event rate for this level of the risk factor versus the reference level

Overall P-value, Test assessing if there is any difference in the event rate across the different levels of the risk factor
